# Development and immunopathological characteristics of an *Alternaria*-induced chronic rhinosinusitis mouse model

**DOI:** 10.1371/journal.pone.0234731

**Published:** 2020-06-16

**Authors:** Seung-Heon Shin, Mi-Kyung Ye, Dong-Won Lee, Mi-Hyun Chae, Sung-Yong Choi

**Affiliations:** Department of Otolaryngology-Head and Neck Surgery, School of Medicine, Catholic University of Daegu, Daegu, South Korea; Forschungszentrum Borstel Leibniz-Zentrum fur Medizin und Biowissenschaften, GERMANY

## Abstract

Airborne fungi are associated with upper and lower airway inflammatory diseases. *Alternaria* is commonly found in nasal secretions and induces the production of chemical mediators from sinonasal mucosa. This study aimed to establish an *Alternaria*-induced chronic rhinosinusitis (CRS) mouse model and determine the influence of host allergic background on the immunopathological characteristics of CRS. BALB/c mice were used for establishing the CRS model. *Alternaria* was intranasally instilled for 8 or 16 weeks with or without ovalbumin (OVA) presensitization. Total serum IgE and *Alternaria*-specific IgE levels were measured by enzyme-linked immunosorbent assay (ELISA). Interleukin (IL)-4, IL-10, interferon (IFN)-γ, and tumor necrosis factor (TNF)-α levels in nasal lavage fluid (NLF) and splenocytes were measured by ELISA and their mRNAs and levels of associated transcription factors in sinonasal mucosa were determined with quantitative reverse-transcriptase polymerase chain reaction (RT-PCR). Hematoxylin-eosin staining and periodic acid-Schiff staining were performed to evaluate histological changes. Total serum IgE was increased in both allergic and non-allergic CRS. IL-4 was strongly expressed in NLF in both allergic and non-allergic CRS at 16 weeks and not only eosinophils but also neutrophils were increased in NLF of non-allergic CRS mice. The levels of Th1, Th2, and Treg cytokines and transcription factor mRNAs were significantly increased in sinonasal mucosa of non-allergic CRS mice. Both inflammatory cell infiltration and goblet cell hyperplasia were increased in CRS mice. Repeated intranasal instillation of *Alternari*a results in sinonasal inflammation with inflammatory cell infiltration. The sinonasal mucosal immune responses against *Alternaria* were shown to differ depending on the host allergic background.

## Introduction

Chronic rhinosinusitis (CRS) encompasses a heterogeneous group of diseases that can be classified as eosinophilic or non-eosinophilic CRS based on the dominant inflammatory cell types. CRS can also be divided into Th1, Th2, and Th17 dominant CRS based on the presence of lymphocyte effector cells in sinonasal tissues. [1] Although the etiology and pathogenesis of CRS are not fully understood, CRS is characterized by the chronic inflammation of sinonasal mucosa with a heterogeneous group of inflammatory responses against allergens, bacteria, fungi, and viruses. CRS had been considered an infectious disease with the focus being placed on identifying the pathogenic microbial organisms. However, recent studies have suggested that CRS may not be related to immune-mediated diseases, the involvement of which may cause the exacerbation of local inflammatory responses.

Fungi are ubiquitous in nature, but relatively few species have been implicated in human diseases. *Alternaria*, *Aspergillus*, *Penicillium*, and *Cladosporium* are commonly found in nasal secretions not only in CRS but also in healthy individuals. [2] Fungi are increasingly recognized as important pathogens in patients with sinusitis; however, their role in the pathogenesis of CRS remains controversial. Fungal components, such as proteins and enzymes, induce immune responses and result in the production of chemical mediators through the interaction of cell membrane receptors. [3, 4] *Alternaria* and *Aspergillus* extracts activate upper and lower airway epithelial cells and enhances the production of several inflammatory mediators; they are considered risk factors for the development of asthma, allergic rhinitis, and CRS. [5–8]

*Alternaria alternata* results in allergic airway inflammation with increased lung expression of type 2 cytokines and eosinophils. [9] In the presence of sinus mucosal trauma, the inoculation of *Alternaria* and *Aspergillus* was shown to induce sinonasal inflammation with inflammatory cell infiltration, epithelial thickening, and goblet cell hyperplasia. [10, 11] Intranasal challenge with *Aspergillus* plus ovalbumin can induce allergic inflammation in sinonasal mucosa with the development of an eosinophilic CRS model. [12] Compare with *Aspergillus*, *Alternaria* strongly enhanced chemical mediatory production from nasal epithelial cells and influenced Th immune responses. [13] Allergic rhinitis and CRS are an inflammatory condition within the sinonasal mucosa. The relationship between allergic rhinitis and CRS is controversial. Nasal allergy related inflammatory mediators have been postulated to develop CRS. [14] However, the evidence linking allergy to CRS is quite low. [15] The aim of this study was to evaluate whether *Alternaria* could induce inflammatory immune responses in sinonasal mucosa and to know if the allergic status of the host could affect the development of fungi induced mucosal inflammation. So we established a CRS mouse model by repeated intranasal instillation of *Alternaria* with or without presensitization by ovalbumin (OVA) and to compare their immunopathological characteristics.

## Materials and methods

### *Alternaria*-induced CRS mouse model

Female BALB/c mice (6 weeks old) were purchased from Hyosung Science Inc. (Daegu, South Korea). They were maintained under standard conditions in a pathogen-free cage. This study was conducted in accordance with the guidelines of the National Institute of Health and was approved by the Institutional Review Board of Animal Experiments of Daegu Catholic University Medical Center (DCIAFCR-180718-11).

**Fig 1:**
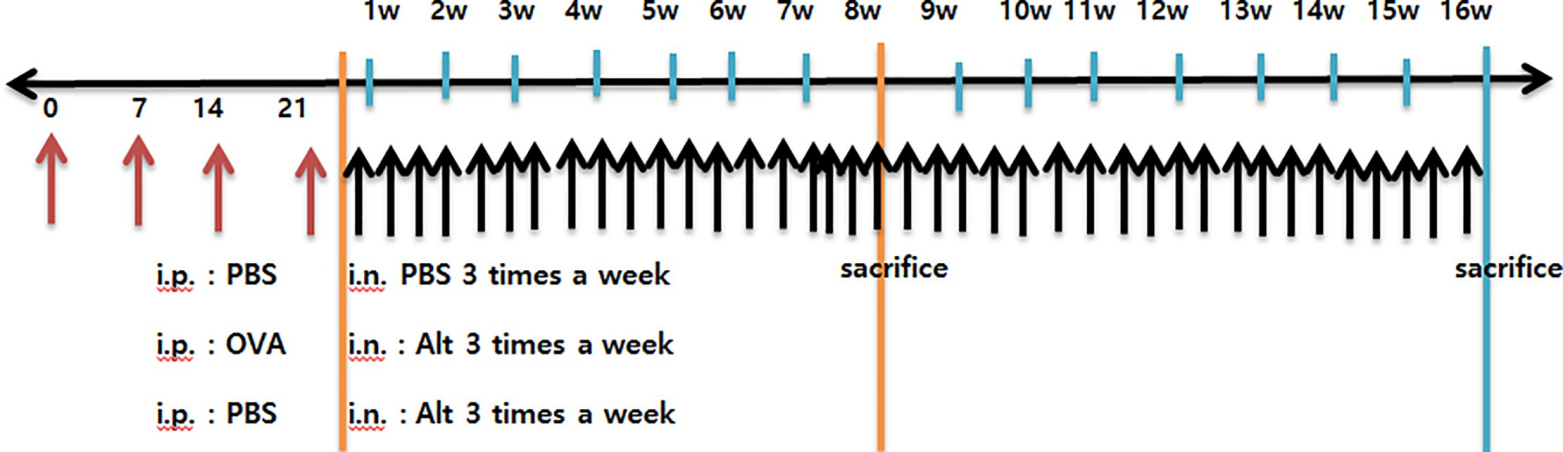
Schematic diagram of *Alternaria* induced chronic rhinosinusitis mouse model. Intraperitoneal injection of ovalbumin (OVA) then intranasal instillation of *Alternaria* for 8 or 16 weeks was designed as an allergic CRS model. Intraperitoneal injections of phosphate buffered saline (PBS) then intranasal instillation of Alternaria for 8 or 16 weeks were designed as non-allergic CRS model. Intraperitoneal injection of PBS then intranasal instillation of PBS for 8 or 16 weeks was designed as negative control mice.

Intraperitoneal injection of ovalbumin (OVA, 75 μg) in 200 μL of phosphate-buffered saline (PBS) containing 2 mg of aluminum hydroxide (Sigma Aldrich, St. Louis, MO, USA) was performed on days 0, 7, 14, and 21 to develop an allergic predisposition. Then, mice were challenged intranasally with 50 μg/mL *Alternaria alternata*; culture filtrate extracts in PBS for 8 or 16 weeks to develop an allergic *Alternaria* CRS model. *Alternaria alternata* extracts (Lot#:312142, Greer Lab, Lenoir, NC, USA) were concentrated, dialyzed, and lyophilized from the medium liquid in which the fungi had been cultivated withextraction of proteins into the media. Intraperitoneal injection of PBS followed by intranasal instillation of *Alternaria* for 8 or 16 weeks was designed to create a non-allergic *Alternaria* CRS model. Intraperitoneal injection of PBS alone followed by intranasal instillation of PBS for 8 or 16 weeks was designed to create negative control mice. Experimental mice were sacrificed 24 hours after the final challenge, as shown in Fig 1.

### Evaluation of chemical mediators in Nasal Lavage Fluid (NLF)

NLF was collected 24 hours after the last challenge at 8 or 16 weeks. A 21-gauge catheter was inserted through a partial tracheal resection site in the direction of the upper airway and into the nasopharynx. One mL of cold PBS was gently perfused into the sinonasal cavity and collected in a tube. NLF was centrifuged at 2,000 rpm for 7 minutes at 4°C. Supernatant was collected to determine the levels of interleukin (IL)-4, IL-10, interferon (IFN)-γ, and tumor necrosis factor (TNF)-α using enzyme-linked immunosorbent assay (ELISA) kits (R&D Systems, Minneapolis, MN, USA). The pellet was resuspended in PBS and stained with May-Grunwald-Giemsa stain and cells differentiated into eosinophils, neutrophils, lymphocytes, and other cells. The average number of cells in five high power field was determined by a well-trained researcher who did not have experimental information on the slides.

### Measurement of serum total IgE

Blood samples were collected from the inferior vena cava 24 hours after the last intranasal challenge. Serum was obtained by centrifugation and total IgE was measured using ELISA (Pharmingen, San Diego, CA, USA).

### Measurement of cytokines and transcription factor mRNA in sinonasal mucosa

Total RNA was extracted from the sinonasal mucosa using Trizol reagent (Invitrogen, Carlsbad, CA, USA). The RNA purity and concentration were measured using a spectrophotometer (Beckman, Mountain View, CA, USA). Complementary DNA was made from 1 μg of RNA using reverse-transcription polymerase chain reaction (RT-PCR) amplification with a PerkinElmer (Norwalk, CT, USA) thermal cycler. From the amplified cDNA, the quantitative polymerase chain reaction was performed using an SYBR Green PCR core kit (PE Applied Biosystems, Foster City, CA, USA). The expression levels of mRNA were measured by the cycle threshold (2^-ΔΔ^CT) method and were normalized to *β-actin*. S1 Table shows the primers used in this study. Initial denaturation was performed at 95°C for 2 minutes, followed by 40 cycles consisting of denaturation at 94°C for 10 seconds, annealing at 60°C for 10 seconds, and elongation at 72°C for 45 seconds. Two technical replicates and 3–4 biological replications were prepared for mRNA study.

### Activation of splenocytes with *Alternaria*

Spleen tissues were isolated from mice and separated into single cells using a 70 μm cell strainer. Red blood cells (RBCs) were removed with RBC lysis buffer (BioLegend, San Diego, CA, USA). The cells were incubated in Roswell Park Memorial Institute-1640 medium supplemented with 10% fetal bovine serum, 100 U/mL penicillin, and 100 μg/mL streptomycin (Gibco, Grand Island, NY, USA). After stimulation with 100 μg/mL *Alternaria* for 72 hours, the supernatant was collected and stored at -70°C until assay. IL-4, IL-10, IFN-γ, and TNF-α level in supernatants were measured using ELISA kits (R&D Systems).

### Histological evaluation of sinonasal mucosa

Mice were painlessly sacrificed with a lethal dose of intraperitoneally administered sodium pentobarbital. Specimens were decalcified in ethylenediaminetetraacetic acid and embedded in paraffin. The tissue was cut into 5-μm-thick coronal sections. Three anatomically similar sections were chosen as in a previous study. [12]

Inflammatory cell infiltration and epithelial thickness were quantified in hematoxylin and eosin-stained sections. The degree of submucosal inflammatory cell infiltration was quantified into four categories as follows (0: none, 1: mild, occasional scattered inflammatory cells, 2: moderate, 3: severe, diffuse infiltration of inflammatory cells). Goblet cell numbers were quantified by Periodic acid-Schiff (PAS) staining at × 200 magnification and the average number of goblet cells was counted using an eyepiece reticle. Epithelial thickness was directly measured at × 400 magnification through a video camera (Olympus Optical Co. Ltd., Tokyo, Japan) and analyzed with DP controller software (ver. 2.2.1.227). All tissue sections were examined blindly with respect to the source of the tissue and counts were determined at three different mucosal areas for each of the three sections per mouse.

### Statistical analysis

All measured parameters are expressed as mean ± standard deviation. One-way analysis of variance followed by Tukey’s test was performed for normally distributed data and the Kruskal-Wallis test with post-hoc Bonferroni-Dunn test was performed for non-normally distributed data (SPSS ver. 21, IBM Corp., Armonk, NY, USA). A probability value of less than 0.05 was considered to represent statistical significance.

## Results

### Total serum IgE level

**Fig 2:**
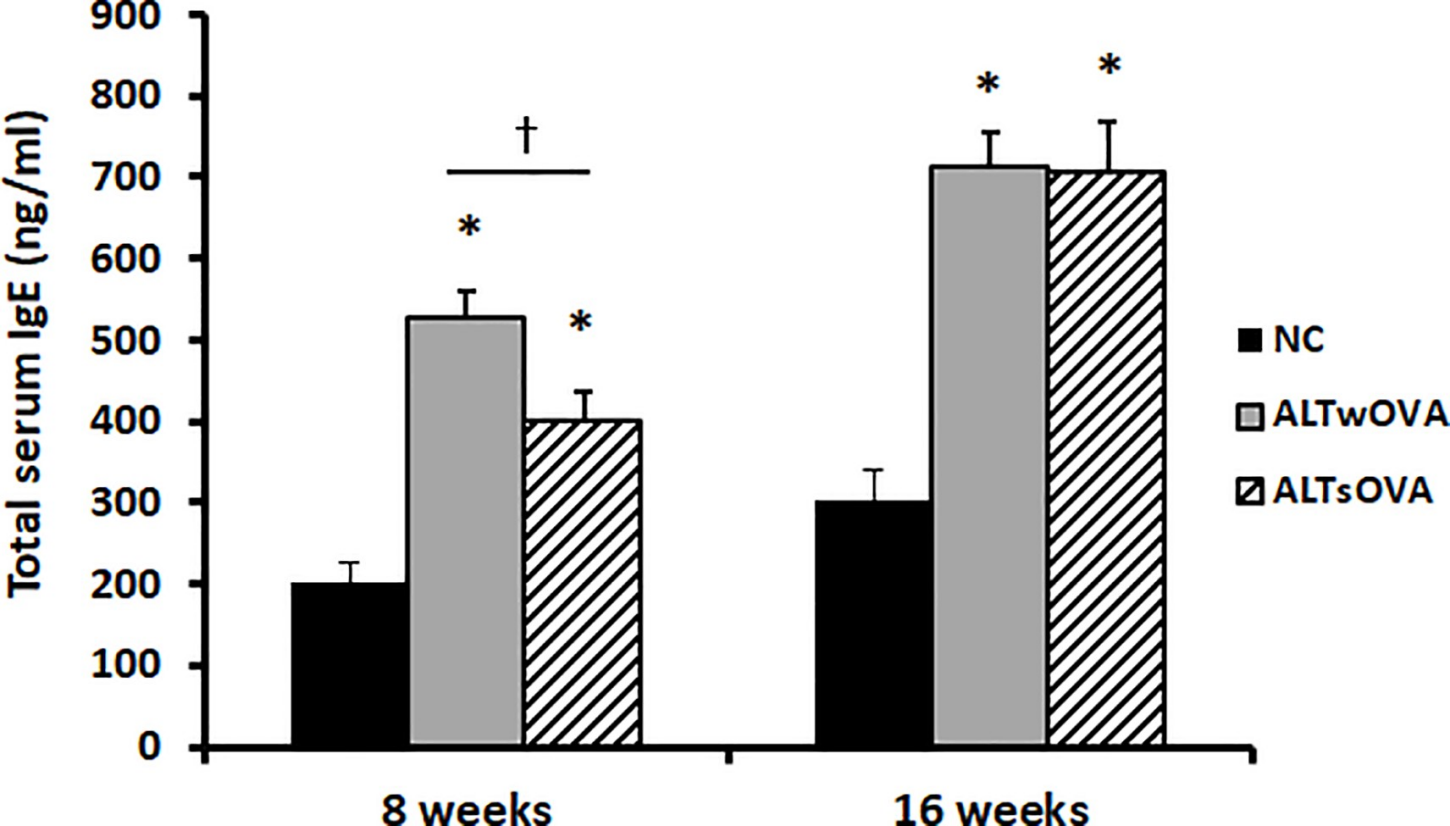
Total serum IgE level at 8 and 16 weeks with repetitive instillation of *Alternaria*. IgE level was significantly increased both allergic and non-allergic mouse model (n = 7 mice per group). NC: negative control, ALTwOVA: *Alternaria* instillation with ovalbumin (OVA) presensitization, ALTsOVA; *Alternaria* instillation without ovalbumin (OVA) presensitization. *p<0.05 vs NC group.

Repeated intranasal instillation of *Alternaria* significantly elevated total serum IgE level regardless of presensitization with OVA at 8 and 16 weeks. Total serum IgE level at 8 weeks was much higher in the presensitized mice (528.2 ± 287.4 ng/mL) than in the non-sensitized group (398.2 ± 160.6 ng/mL), but these groups’ levels became similar after 16 weeks (712.3 ± 171.3 ng/mL with presensitization, 704.3 ± 431.6 ng/mL without presensitization) (Fig 2).

### Chemical mediators and inflammatory cells in NLF

**Fig 3:**
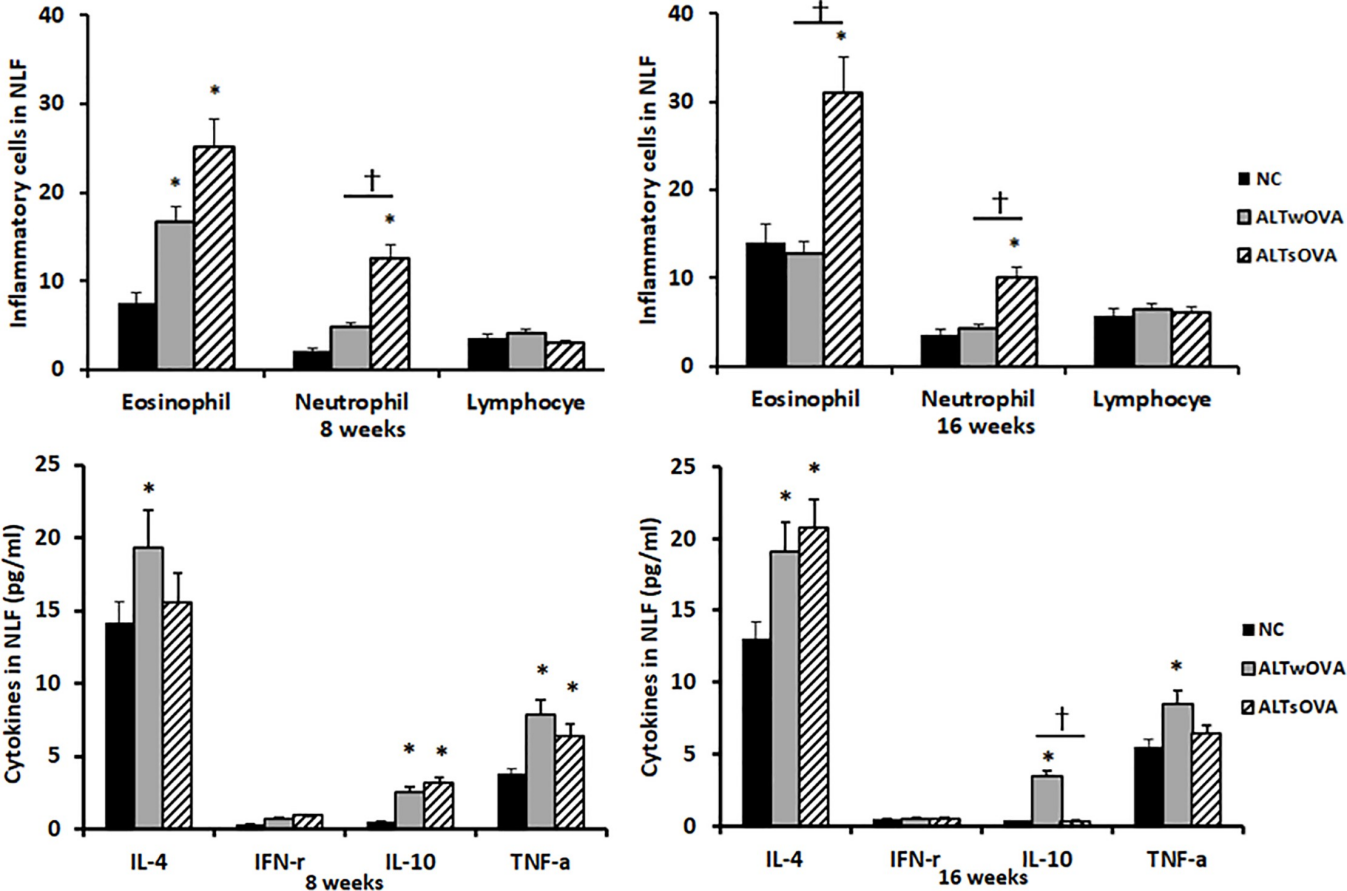
Inflammatory cell differentials and chemical mediator levels in Nasal Lavage Fluid (NLF) of the *Alternaira* induced chronic rhinosinusitis mouse model. Eosinophil and neutrophils were increased in NLF of non-allergic mouse model. Th2, Treg, and inflammatory cytokine revel were strongly expressed in NLF of allergic mouse model (n = 7 mice per group). NC: negative control, ALTwOVA: *Alternaria* instillation with ovalbumin (OVA) presensitization, ALTsOVA; *Alternaria* instillation without ovalbumin (OVA) presensitization. *p<0.05 vs NC group, †p<0.05 vs with or without OVA group.

Repeated intranasal instillation of *Alternaria* elevated the level of IL-4, IL-10, and TNF-α in NLF of allergic CRS mice at 8 (IL-4, 19.4 ± 9.6 pg/mL; IL-10, 2.6 ± 2.3 pg/mL; TNF-α, 7.9 ± 5.9 pg/mL) and 16 weeks (IL-4, 19.1 ± 5.9 pg/mL; IL-10, 3.5 ± 1.7 pg/mL; TNF-α, 8.5 ± 5.2 pg/mL). Without presensitization with OVA, the elevated IL-10 level of 3.2 ± 2.5 pg/mL at 8 weeks became as low as 0.4 ± 0.2 pg/mL at 16 weeks. INF-γ level was not significantly influenced by the intranasal instillation of *Alternaria* (Fig 3).

When the mice were treated with *Alternaria* for 8 and 16 weeks, eosinophil and neutrophil counts were significantly increased in NLF of non-allergic CRS mice. Only the level of eosinophils was increased at 8 weeks in NLF of OVA-sensitized mice, compared with that in negative control mice. The levels of lymphocytes in NLF did not differ significantly among the three groups (Fig 3).

#### Chemical mediator production from splenocytes

**Fig 4:**
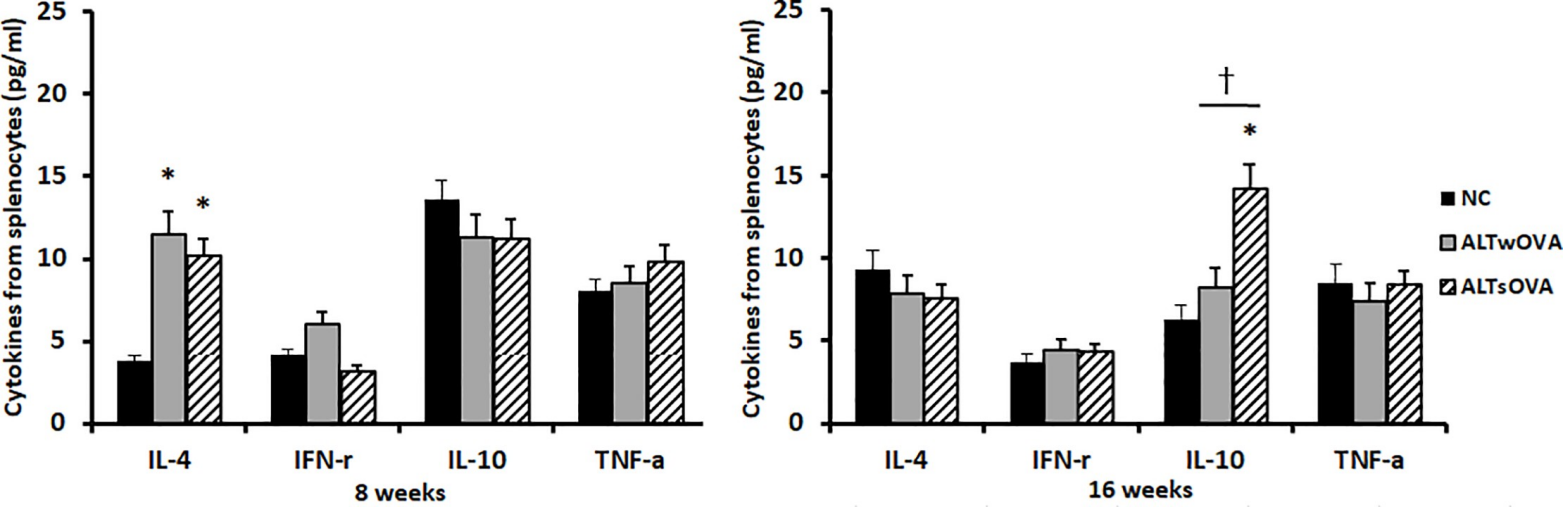
Chemical mediator production from splenocyte after stimulated with 100 ug/mL of *Alteranria* for 72 hours. IL-4 production was significantly increased in allergic and non-allergic mica at 8 weeks and IL-10 production was significantly increased in non-allergic mice (n = 7 mice per group). NC: negative control, ALTwOVA: *Alternaria* instillation with ovalbumin (OVA) presensitization, ALTsOVA; *Alternaria* instillation without ovalbumin (OVA) presensitization. *p<0.05 vs NC group, †p<0.05 vs with or without OVA group.

Mouse spleen cells were stimulated with *Alternaria* for 72 hours. IL-4 production was significantly increased in allergic (11.5 ± 4.7 pg/mL) and non-allergic (10.2 ± 5.6 pg/mL) CRS mice compared with that in the control group (3.8 ± 2.7 pg/mL) at 8 weeks. However, the levels of IL-10, IFN-γ, and TNF-α production did not differ significantly among the three groups. In intranasal instilled mice at 16 weeks, only IL-10 production was significantly increased in non-allergic CRS mice (14.3 ± 7.3 pg/mL) compared with that in the other groups (sensitized group, 8.21 ± 6.5 pg/mL; negative control group, 6.3 ± 3.7 pg/mL) (Fig 4).

### mRNA expression of sinonasal mucosal cytokines and transcription factors

**Fig 5:**
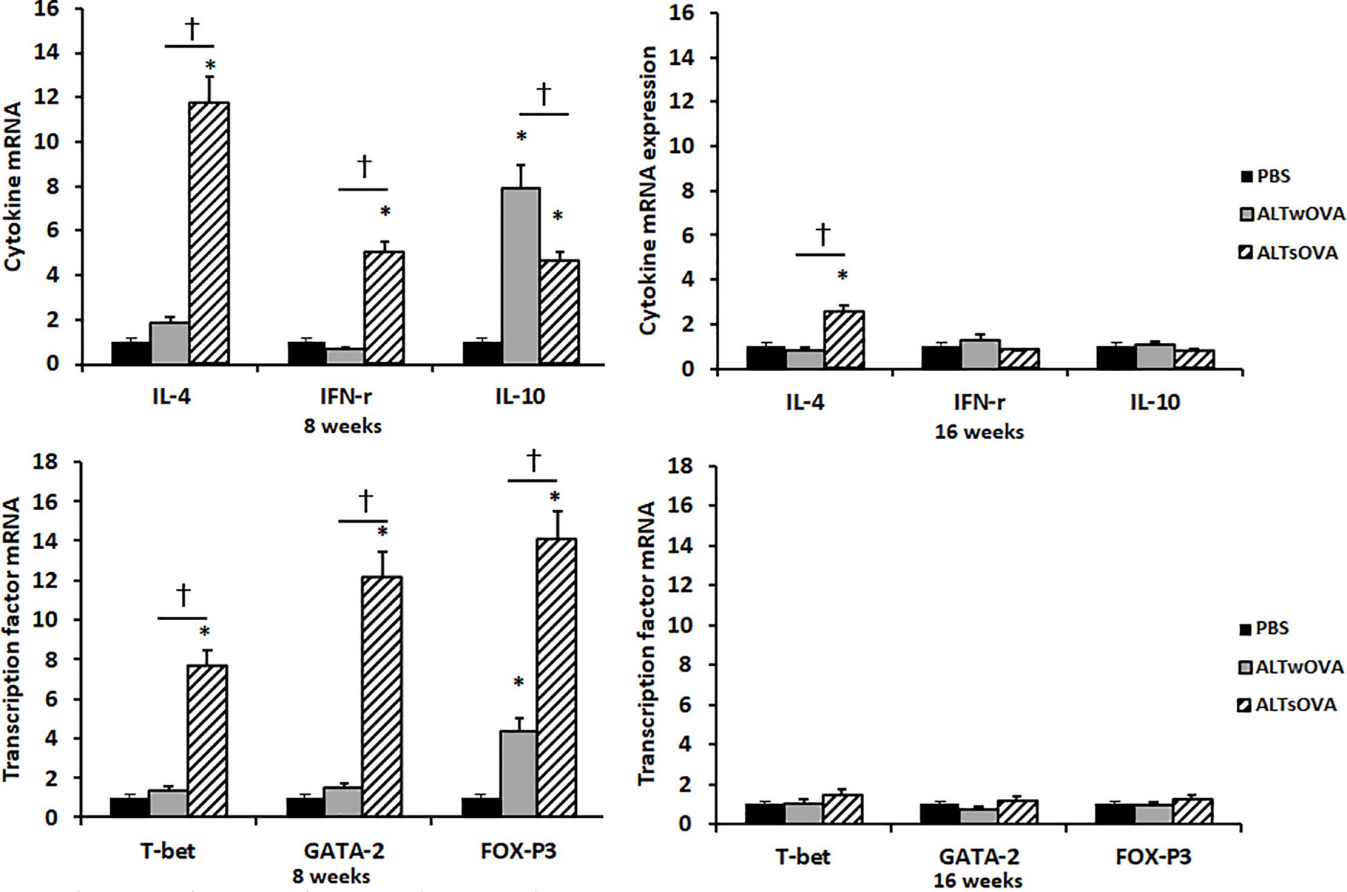
Chemical mediator mRNA and transcription factor expression in sinonasal mucosa of *Alternaria* induced Chronic RhinoSinusitis (CRS) mouse model. IL-4 and IFN-γ and their transcription factor mRNA expression were significantly increased non-allergic CRS group at 8 weeks. IL-10 and its transcription factor mRNA expression were significantly increased both allergic and non-allergic CRS group at 8 weeks (3–4 mice per group). NC: negative control, ALTwOVA: *Alternaria* instillation with ovalbumin (OVA) presensitization, ALTsOVA; *Alternaria* instillation without ovalbumin (OVA) presensitization. *p<0.05 vs NC group, †p<0.05 vs with or without OVA group.

We performed real-time RT-PCR to determine the effect of the intranasal instillation of *Alternaria* on the mRNA expression of Th-related cytokine and T-cell subset transcription factors in sinonasal mucosa. After 8 weeks of instillation with *Alternaria*, IL-10 and FOXp3 mRNA expression levels were significantly increased in both allergic and non-allergic CRS mice. The mRNA expression of IL-4, INF-γ, T-bet, and GATA3 was significantly increased by *Alternaria* in non-allergic CRS mice compared with that in allergic CRS and negative control mice. After 16 weeks of instillation with *Alternaria*, only IL-4 mRNA expression was significantly increased in the non-allergic group compared with that in the allergic and negative control groups (Fig 5).

### Histopathological characteristics

**Fig 6:**
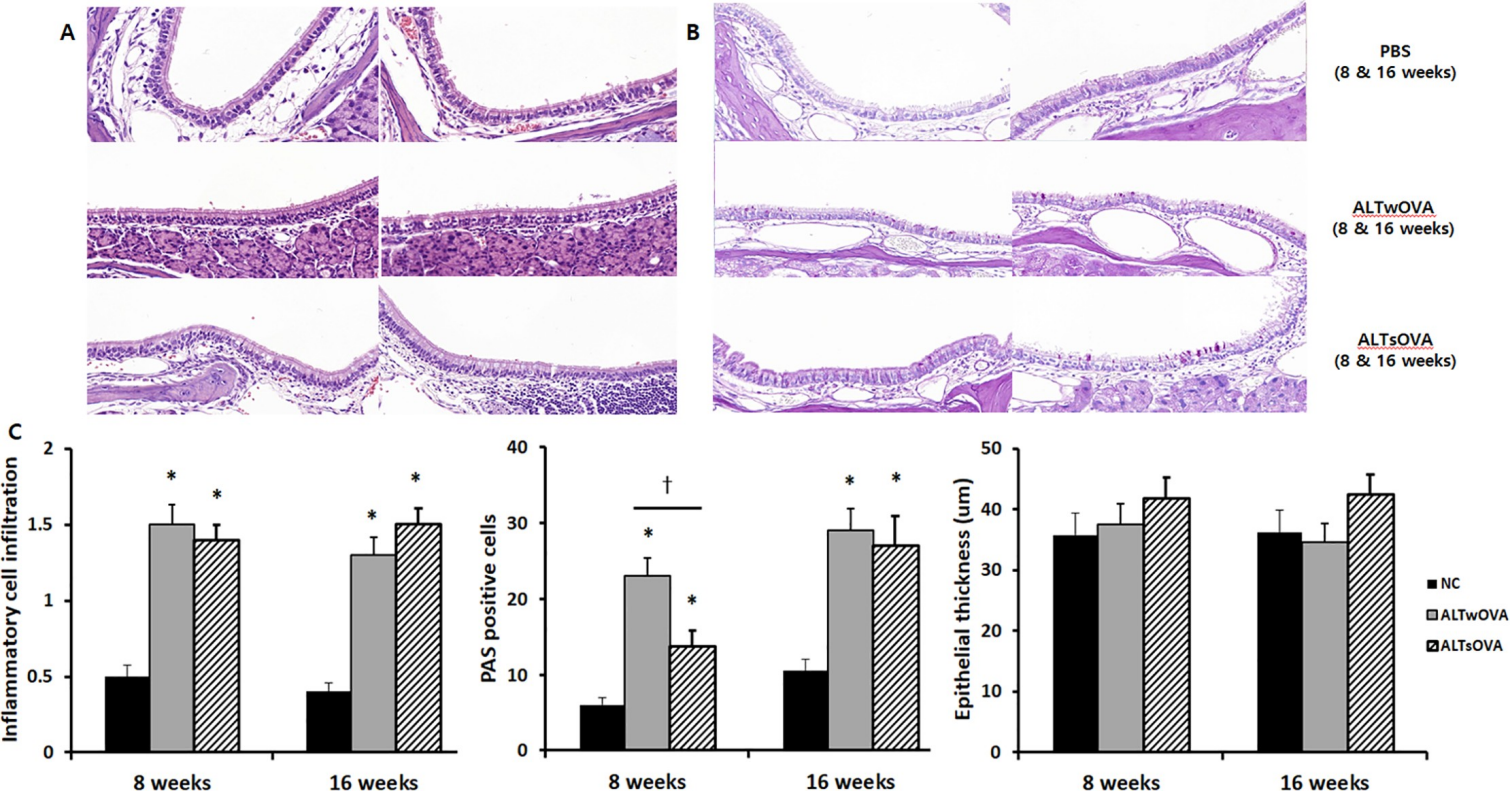
Histologic characteristics of the *Alternaira* induced Chronic RhinoSinusitis (CRS) mouse model. Inflammatory cell infiltration (A, x400) and periodic acid-Schiff (PAS) positive cells (B, x400) were significantly increased in sinonasal mucosa of allergic and non-allergic CRS mouse model of the *Alternaira* induced chronic rhinosinusitis mouse model (3–4 mice per group). NC: negative control, ALTwOVA: *Alternaria* instillation with ovalbumin (OVA) presensitization, ALTsOVA; *Alternaria* instillation without ovalbumin (OVA) presensitization. *p<0.05 vs NC group, †p<0.05 vs with or without OVA group.

The intranasal instillation of *Alternaria* significantly increased inflammatory cell infiltration of the submucosal area with or without OVA sensitization at 8 (1.5 ± 0.6 and 1.4 ± 0.5 with or without sensitization) and 16 weeks (1.3 ± 0.3 and 1.5 ± 0.5 with or without sensitization), compared with that in the control group (0.5 ± 0.3 and 0.4 ± 0.3 at 8 and 16 weeks) (Fig 6).

PAS-positive goblet cells in sinonasal mucosa were significantly increased in both allergic and non-allergic CRS groups at 8 (23.0 ± 13.4 and 13.8 ± 7.5 respectively) and 16 weeks (29.0 ± 9.7 and 27.0 ± 13.3 respectively) compared with that in the control group (6.0 ± 2.5 and 10.5 ± 3.4 at 8 and 16 weeks). Although epithelial thickness tended to increase in the *Alternaria*-instilled groups, there was no significant difference among the three groups (Fig 6).

## Discussion

Fungi are environmentally ubiquitous and commonly found in airway secretions of healthy and CRS patients. [1, 16] Protease activity form *Alternaria* induces proinflammatory cytokines and thymic stromal lymphopoietin (TSLP) production from respiratory epithelial cells and commonly associated with CRS. [17, 18] Epithelial cells derived TSLP and IL-33 secretion into the airway induce Th2-type inflammation. [17, 19] In this study, we tried to determine the immunologic effect of *Alteranria alternata* on sinonasal mucosa inflammation and the immunopathologic characteristics of *Alternaria* induced CRS mouse model. Repetitive intranasal stimulation with *Alternaria* results in prominent sinonasal inflammation regardless allergic predisposition. The association between allergy and CRS remains debatable and nearly an equal number of studies supported or refuted an association of allergy with both CRS with nasal polyps or CRS without nasal polyps. [20] According to our data, *Alternaria* could develop sinonasal inflammation regardless of allergic host background and *Alternaria* induced Th2 dominant immune response with allergic predisposition, whereas Th1 and Th2 immune responses without allergic predisposition. Fungal spores are continuously inhaled and deposit in sinonasal mucosal and influence mucosal immune responses. However, we used culture filtrated extracts of *Alternaria* to develop CRS mouse model, so we need further study to determine the immunopathologic characteristics of *Alternaria* spore induced CRS mouse.

*Alternaria* develops an allergic airway response characterized by increased Th2 cytokine and eosinophil infiltration and high level of serum IgE. [21] Intranasal instillation of *Alternaria* caused the high level of serum IgE in regardless of allergic status. Eosinophil count was increased in NLF by intranasal instillation of *Alternaria* at 8 weeks, in regardless of allergic status. However, at 16 weeks, eosinophils only increased in non-allergic CRS mouse. And neutrophils are only increased in non-allergic CRS mouse. Sinonasal mucosal inflammatory cell infiltration was not significantly influenced the allergic status of mice. These findings suggest that the intranasal instillation of *Alternaira* can induce allergic responses with high serum total IgE, tissue inflammatory cell infiltration, and mucus production regardless of allergic status. However, the eosinophilic inflammatory responses seem to more dominant in allergic CRS mouse and both eosinophilic and neutrophilic inflammatory responses seem to happen without allergic predisposition.

Intranasal instillation of *Alternaria* increased IL-4 concentration in NLF at 8 and 16 weeks in allergic CRS mouse. Although IL-4 was not statistically significantly increased at 8 weeks, the increasing pattern of IL-4 in NLF was similar in non-allergic CRS mouse. However, when the splenocytes were stimulated with *Alternaria*, IL-4 was significantly increased at 8 weeks but not 16 weeks. These results may be due to the intranasal installation induce early local and systemic Th2 inflammatory responses, and over time, the systemic Th2 inflammatory response was weakened with continuous Th2 local inflammatory responses. Treg cytokine, IL-10 was increased in NLF of allergic CRS mice at 8 and 16 weeks and from splenocytes in non-allergic CRS mouse at 16 weeks. These findings may indicate that intranasal instillation of *Alternaria* show different local and systemic immune responses. After 8 weeks of intranasal instillation developed local and systemic Th1 and/or Th2 inflammation. However, after 16 weeks, local inflammatory immune responses diminished with increased systemic immune tolerance with increased production of IL-10. According to the previous studies, CRS mouse models developed inflammatory immune response after 4 to 12 weeks of stimulation with single pathogens. [10, 12, 22] When the CRS mouse model was made with multiple airborne allergens, sinonasal inflammation maintained for more than 12 weeks. [23] We presume that regional exposure with single allergens may induce local and systemic inflammatory immune responses initially, then local inflammatory responses may decrease with increased systemic immune tolerance. To maintain or aggravate the local and systemic inflammation, another pathogenic stimulation may be needed. The role of fungi in pathogenesis in CRS is still controversial. However, if the sinonasal fungal inflammation goes with a bacterial or viral infection, the local and systemic inflammations may persist and aggravate for a longer period of time. Respiratory epithelial cells are the first mucosal cells exposed to the environmental stimuli, such as airborne allergens, fungi, viruses, and bacteria. If the innate immune defense against pathogens is destroyed by some reason, these pathogens may interact to aggravate local and systemic inflammation. To determine the differences in the immune responses against *Alternaria*, we need to evaluate local and systemic inflammatory patterns at different time intervals and compare the expressions of other inflammatory and suppressive cytokines in sinonasal mucosa.

Sinonasal mucosal cytokine and their transcription factor mRNA expression pattern was different from cytokine concentration in NLF. Th1 and Th2 cytokine and their transcription factor mRNA were significantly increased in non-allergic CRS mouse and Treg cytokine and transcription factor mRNA was significantly increased regardless of allergic predisposition. The exact reason could not explain with this study, but there are some possibilities. Sinonasal mucosal mRNA level could represent protein concentration of sinonasal mucosa but not the concentration in NLF. Unfortunately, we did not measure the protein level of Th cytokines and directly compare mRNA and protein levels in mucosa. The IL-4 and transcription factor mRNA expression was not statistically significant increase in allergic CRS mouse. However, IL-4 and its transcription factor mRNA expression tended to increase with significant increase of IL-4 in NLF. These results may associate with time interval difference between mRNA expression, protein production, and their secretion to nasal cavity.

## Conclusion

Repetitive intranasal instillation of *Alternari*a results in Th2 and eosinophil dominant immune response in allergic predisposition state. Without allergic background, repetitive stimulation of *Alternaria* results in not only Th2 but also Th1 and Treg immune response with eosinophil and neutrophil dominant inflammation. Based on these results, intranasal instillation of *Alternaria* can induce sinonasal mucosa inflammation with Th immune responses. And the sinonasal mucosal immune responses against *Alternaria* were different based on the differences host allergic background and different time intervals.

## Supporting information

S1 Table Primers used in the experiments. (DOCX)

S1 Data (XLS)

S2 Data (XLS)

S3 Data (XLS)
