# Supplementary material for: Development and immunopathological characteristics of an Alternaria-induced chronic rhinosinusitis mouse model
Source: PLoS One. 2020 Jun 16;15(6):e0234731. doi: 10.1371/journal.pone.0234731 (PMC7297365; doi:10.1371/journal.pone.0234731)
Supplement: S1 Table — (DOCX) [file pone.0234731.s001.docx]

S1 **Table. Primers used in the experiments**

| **Primer** | **Sequence** | **Bp** | **Gene number** |
| --- | --- | --- | --- |
| **IL-4** | **Forward 5’- CAATTGCAATGCCATCTACAGGAC-3’**  **Reverser 5’-TTTTGGTATCGGGGAGGCTG-3’** | **104** | **NM_021283.** |
| **IL-10** | **Forward 5’-GCCAGAGCCACATGCTCCTA-3’**  **Reverse 5’-GATAAGGCTTGGCAACCCAAGTAA-3’** | **145** | **NM_010548.** |
| **INF-γ** | **Forward 5’-CGGCACAGTCATTGAAAGCCTA-3’**  **Reverse 5’-GTTGCTGATGGCCTGATTGTC-3’** | **199** | **NM_008337.** |
| **T-bet** | **Forward 5’-GCCAGGGAACCGCTTATA-3’**  **Reverse 5’-CCTTGTTGTTGGTGAGCTTTA-3’** | **104** | **NM_019507.** |
| **GATA-3** | **Forward 5’-TACCACCTATCCGCCCTATG-3’**  **Reverse 5’-GCCTCGACTTACATCCGAAC-3’** | **101** | **NM_001355110** |
| **Foxp3** | **Forward 5’-CACCTATGCCACCCTTATCCG-3’**  **Reverse 5’-CATGCGAGTAAACCAATGGTAGA-3’** | **91** | **NM_001199347** |
| **β-actin** | **Forward 5’-GCAGAAGGAGATTACTGCTCT-3’**  **Reverse 5’- GCTGATCCACATCTGCTGGAA-3’** | **136** | **NM_007393.** |
